# Population-based incidence, seasonality and serotype distribution of invasive salmonellosis among children in Nanoro, rural Burkina Faso

**DOI:** 10.1371/journal.pone.0178577

**Published:** 2017-07-10

**Authors:** Issa Guiraud, Annelies Post, Seydou Nakanabo Diallo, Palpouguini Lompo, Jessica Maltha, Kamala Thriemer, Christian Marc Tahita, Benedikt Ley, Karim Derra, Emmanuel Bottieau, Adama Kazienga, Céline Schurmans, Raffaella Ravinetto, Eli Rouamba, Johan Van Griensven, Sophie Bertrand, Halidou Tinto, Jan Jacobs

**Affiliations:** 1 IRSS/Clinical Research Unit of Nanoro (CRUN), Nanoro, Burkina Faso; 2 Department of Microbiology and Immunology, University of Leuven (KU Leuven), Leuven, Belgium; 3 Department of Clinical Sciences, Institute of Tropical Medicine (ITM), Antwerp, Belgium; 4 Centre Muraz, Bobo-Dioulasso, Burkina Faso; 5 Center for Molecular and Vascular Biology, University of Leuven (KU Leuven), Leuven, Belgium; 6 Menzies School of Health Research, Darwin, Australia; 7 Department of Public Health, Institute of Tropical Medicine (ITM), Antwerp, Belgium; 8 Institute of Public Health, Brussels, Belgium; University of California, Davis, UNITED STATES

## Abstract

**Background:**

Bloodstream infections (BSI) caused by *Salmonella* Typhi and invasive non-Typhoidal *Salmonella* (iNTS) frequently affect children living in rural sub-Saharan Africa but data about incidence and serotype distribution are rare.

**Objective:**

The present study assessed the population-based incidence of *Salmonella* BSI and severe malaria in a Health and Demographic Surveillance System in a rural area with seasonal malaria transmission in Nanoro, Burkina Faso.

**Methods:**

Children between 2 months—15 years old with severe febrile illness were enrolled during a one-year surveillance period (May 2013—May 2014). Thick blood films and blood cultures were sampled and processed upon admission. Population-based incidences were corrected for non-referral, health seeking behavior, non-inclusion and blood culture sensitivity. Adjusted incidence rates were expressed per 100,000 person-years of observations (PYO).

**Results:**

Among children < 5 years old, incidence rates for iNTS, *Salmonella* Typhi and severe malaria per 100,000 PYO were 4,138 (95% Confidence Interval (CI): 3,740–4,572), 224 (95% CI: 138–340) and 2,866 (95% CI: 2,538–3,233) respectively. Among those aged 5–15 years, corresponding incidence rates were 25 (95% CI: 8–60), 273 (95% CI: 203–355) and 135 (95% CI: 87–195) respectively. Most iNTS occurred during the peak of the rainy season and in parallel with the increase of *Plasmodium falciparum* malaria; for *Salmonella* Typhi no clear seasonal pattern was observed. *Salmonella* Typhi and iNTS accounted for 13.3% and 55.8% of all 118 BSI episodes; 71.6% of iNTS (48/67) isolates were *Salmonella* enterica serovar Typhimurium and 25.4% (17/67) *Salmonella* enterica serovar Enteritidis; there was no apparent geographical clustering.

**Conclusion:**

The present findings from rural West-Africa confirm high incidences of *Salmonella* Typhi and iNTS, the latter with a seasonal and *Plasmodium falciparum*-related pattern. It urges prioritization of the development and implementation of *Salmonella* Typhi as well as iNTS vaccines in this setting.

## Introduction

Invasive *Salmonella* infections (Typhoidal and non-Typhoidal) cause a huge burden of disease, especially in resource-limited settings. Unlike *Salmonella* Typhi which occurs in previously healthy individuals, invasive non-Typhoidal *Salmonella* (iNTS) infection appears to be predominantly associated with host-related factors including malnutrition and malaria in children, and HIV in children as well as adults [1]. The estimated prevalence of typhoid fever in Low and Middle Income Countries was 11.9 million (95% CI 9.9–14.7) cases with 129,000 (95% CI 75,000–208,000) deaths in 2010 [2]. For iNTS the prevalence is estimated to be lower, at 3.4 million cases per year (95% CI 2.1–6.5), but with a higher case fatality rate of 20–30% [3]. An estimated 681,316 (95% CI 415,164–1,301,520) patients die annually as a result of iNTS in Sub-Saharan Africa alone, the majority of which are children < 5 years old [4]. However, these estimations are based on limited data from in-hospital surveillance as population-based incidence rates are rare. This can largely be attributed to the lack of microbiological facilities in rural African settings [5].

The Clinical Research Unit of Nanoro (CRUN), Burkina Faso has established a Health and Demographic Surveillance System (HDSS) since 2009, allowing the measurement of incidences of health-related events [6]. We conducted a prospective non-interventional study to assess the population-based incidence, seasonality, serotype distribution, and geographic clusters of iNTS and *Salmonella* Typhi BSI among febrile and severely ill children. Additionally we assessed the population-based incidence of severe malaria among enrolled children.

## Methods

### Study site

Burkina Faso is a low income country in western Africa with a population of 18 million, ranking 183/188 on the Human Development Index of 2015 [7]. The Nanoro HDSS is located in the province of Boulkiemdé, in the Center-West region of Burkina Faso, 90 km away from the capital Ouagadougou. It is located within the Health District of Nanoro and covers an area of 594.3 km^2^. The HDSS catchment area comprises 36% of the Nanoro Health district, encompassing 24 villages with a population of about 60,000 inhabitants of whom 20% are children less than five years of age [6]. About 90% of the population is engaged in subsistence agriculture. Malaria transmission (mainly *Plasmodium falciparum*) is hyper-endemic from July—October corresponding to the rainy season [8]. In 2010 the overall under-5 mortality rate was 142/1.000 life-births in the Centre-West of Burkina Faso [9]. The HIV-prevalence in this area was 0.9% among pregnant women in 2013 [10]. The national Extended Program of Immunization includes, amongst others, *Haemophilus influenzae* type b since January 2006 and the 13-valent Pneumococcal Conjugate Vaccine (PCV-13) since October 2013; typhoid vaccination is not routinely done. Malaria control programs in place during the study period included the distribution of bed nets among families.

### Enrollment sites, period and inclusion criteria

The Nanoro HDSS encompasses 7 Healthcare Centers (HC) with to one district referral hospital “Centre Médical avec Antenne Chirurgicale (CMA)”. Typically HCs provide first line care. All HCs have an observation ward (3–6 beds) where patients can be observed for a maximum of one day. Patients in critical condition or with warning signs are referred to CMA.

Enrollment was performed at 2 sites: the Pediatric ward (36 beds) of CMA and the Healthcare Center of Nazoanga. HC Nazoanga is the largest HC in the HDSS and located at 14 km distance of CMA. Due to its function as a reference hospital, patients enrolled at CMA include patients living inside and outside the HDSS catchment area.

All children between 2 months and 15 years of age (representing the most vulnerable patient group) presenting at either site between May 13^th^ 2013 and May 12^th^ 2014 were screened for eligibility.

Patients were eligible for inclusion if they had (i) fever (axillary temperature ≥ 37.5°C or reported history of fever in the past 48 hours) or temperature ≤ 35.5°C (ii) or suspicion of severe localized bacterial infection such as pneumonia, arthritis, osteomyelitis and/or soft tissue infection, peritonitis, meningitis, or complicated urinary tract infection. Clinical examination was performed by trained nurses trough a detailed case report form and under the supervision of the study principal investigator (a medical doctor) and a qualified pediatrician. Basic demographic data including age, gender and geographic origin as well as medical history, reported prior antibiotic (48 h) or antimalarial (2 weeks) treatment and presumptive diagnosis upon admission were recorded. Discharge and in-hospital mortality were recorded.

### Laboratory, sampling and analysis

The clinical laboratory of CRUN is situated on the same compound as CMA. Laboratory practices have been standardized using Standard Operational Procedures (SOPs) and a quality management system according to Good Clinical Laboratory Practices principles is implemented.

Upon inclusion venous blood was sampled for malaria microscopy, full blood count (Sysmex XS1000i (Sysmex Corporation, Kobe, Japan)) and blood cultures (1–3 ml pediatric culture bottle (BD BACTEC Peds PlusTM /F, (Becton Dickinson and Company, Sparks, Maryland, USA)) as previously described [11]. Malaria was diagnosed by microscopy [12] and, at HC Nazoanga, also by rapid diagnostic test (RDT, SD Bioline Antigene Pf (Standard Diagnostics, Hagal-Dong, Korea)).

Samples from CMA were transported to CRUN within 15 minutes after collection. For HC Nazoanga, samples were stored and transported to CRUN by car (ambient temperature for blood cultures, 2–8°C for EDTA-anticoagulated blood) at the end of each day and transport time was recorded. The quality indicators are reported in S1 Appendix.

### Rainfall data

Data on rainfall were collected from “Zone d’Appui Technique” in Nanoro, which records rainfall data (mm of precipitation) on a monthly basis.

### Case definitions

Bloodstream Infection (BSI) was defined as growth of pathogens from blood culture. Non-pathogenic bacteria or skin flora including coagulase-negative staphylococci, *Bacillus* spp., *Corynebacterium* spp. and *Propionibacterium* spp. were considered contaminants. Malaria was defined as the presence of asexual *P*. *falciparum* parasites by microscopy. Severe malaria was defined as confirmed malaria in presence of at least one of the World Health Organization criteria for severe malaria [13] (*i*.*e*. severe anemia—(hemoglobin concentration < 5 g/dl), hyperparasitemia (≥ 4%), coma or impaired consciousness, respiratory distress, icterus, hemoglobinuria or seizures). In children < 5 years old, severe acute malnutrition was defined as a mid-upper arm circumference less than 115 millimeters [14].

### Quality assurance, data management and statistical analysis

For the duration of the study compliance to good clinical (laboratory) practices was monitored by the Clinical Trials Unit of the Institute of Tropical Medicine, Antwerp (ITM). Trial Management Group Meetings were organized once a month. Data quality was monitored and bi-monthly status reports were provided by the principal investigator. Clinical and laboratory data were entered twice into an Epi-InfoTM^7^ database (CDC, Atlanta, Georgia, USA). After conformity check, statistical analysis was done with Stata 12 (Stata Corp., College Station, TX, USA). To assess differences in proportions, the Chi-square or two-tailed Fisher’s exact test were used. Mean and median values were compared using the Student’s t-test and the Wilcoxon Mann–Whitney non-parametric test respectively. A *p*-value < 0.05 was considered significant.

### Crude incidence rates of invasive salmonellosis and severe malaria

Population-based incidence rates were calculated for patients residing in the HDSS area. Only the first isolate per patient was considered, except if subsequent sampling had been done more than two weeks after the first sampling. For calculation of the crude incidence rates, the total and age-specific numbers of cases were used as the numerator and the respective person-year of observations (PYO) as the denominator; 95% confidence intervals (CI) were calculated using the Wilson score method. Incidence rates were calculated per 100,000 PYO and expressed for (i) children aged 2 months– 5 years (further referred to as “< 5 years old group”) and (ii) children aged 5–15 years (further referred to as “5–15 years old group”).

### Correction factors used to calculate the adjusted incidence rates

Crude incidences were converted to adjusted incidences by correcting for the following factors: (i) non-referral from Healthcare Centers to CMA, (ii) seeking healthcare elsewhere than in CMA or Health Centers, (iii) non-enrollment of eligible patients, (iv) sensitivity of blood cultures combined with estimated loss of sensitivity due to under-filling of the bottles. For correction of the incidence of severe malaria, only correction factors (ii) and (iii) were considered.

(i) Correction for non-referral to CMA: Only patients with acute warning signs are referred to CMA hospital whereas those with a milder clinical presentation are treated at the HC level. Based on statistics from the District Health Office complemented with surveillance data from HC, Nazoanga the referral proportion of children with febrile illness was estimated at 25% [8].

(ii) Correction for seeking healthcare elsewhere than in Healthcare Centers and CMA: A Healthcare Utilization Survey (HUS) was carried out among a sample of households in the HDSS area in order to map the healthcare utilization of children in case of fever. HUS methods were based on those previously published by the Typhoid Fever surveillance in Africa Program consortium (TSAP) [15]. A total of 160 households (1,615 patients) were visited as part of the routine HDSS visits one to four times over a period of 18 months. All reported episodes of fever among household members during the three months prior to the visit and their corresponding healthcare seeking behavior were recorded. Based on the obtained data a correction factor for healthcare-seeking behavior was calculated.

(iii) Correction for non-enrollment of eligible children comprised not giving consent, leaving CMA or HC Nazoanga before sampling as well as patients who were not sampled because of lack of blood culture bottles.

(iv) Correction for sensitivity of blood culture and inadequate filling: Under-filling and over-filling of blood cultures was defined as filling below or above the recommended volume of 1–3 ml. To estimate the impact of under-filling, the volume lost by under-filling was calculated by subtracting the actual median volume of the under-filled bottles from the minimum volume required (= 1.0 ml). Next, this difference was multiplied by the proportion of under-filled bottles. Finally the correction for sensitivity of blood culture was applied. The age groups of < 5 years old and 5–15 years old were considered separately.

A detailed description of the methodology and results of the HUS and a brief literature review about the sensitivity of blood cultures can be found in S2 Appendix.

### Ethics statement

The study was conducted according to the principles expressed in the Declaration of Helsinki [16] and the WHO Good Clinical Practices Guidelines (WHO 1995), and was approved by the national ethics committee of Burkina Faso (Comité d’Ethique pour la Recherche en Santé, deliberation N°2013-01-08 from January 09^th^ 2013), the institutional review board of the ITM, Antwerp (Reference 843/12 from December 4^th^, 2012) and the ethics committee of the University Hospital of Antwerp (January 01^st^, 2013). Written informed consent was obtained from parents/guardians prior to inclusion, in presence of an independent witness in case of illiteracy.

## Results

### Patient enrollment

In total 1,339 children were enrolled, representing 91.6% of 1,461 eligible children. Table 1 summarizes the breakdown of patients and samples. Patients were equally distributed over CMA and HC Nazoanga. Age and gender distribution were similar for both study sites. Most non-enrollments of eligible patients (106/122, 86.9%) occurred in HC Nazoanga. There were more cases of severe malaria enrolled in CMA (198/691, 28.6%) than in HC Nazoanga (54/648, 8.3%), p < 0.001. BSI cases were also more frequent in CMA than in HC Nazoanga (31/648, 4.8% versus 87/691, 12.6%, p < 0.001).

**Table 1:** Breakdown of patients and samples for the total study area (in- and outside Health Demographic Surveillance System (HDSS) and both study sites.

	Patients from HDSS catchment area		Patients from outside HDSS area	Total
	HC Nazoanga	CMA	CMA	
Patients eligible, n	768	369	324	1,461
Patients enrolled, n (% of patients eligible)	648 (84.4)	367 (99.5)	324 (100)	1,339 (91.6)
Age (in month) of patients enrolled, median (IQR)	25.2 (12.3–47.3)	21.3 (10.9–42.7)	29.1 (14.1–50.6)	24.6 (12.2–46.4)
Male/Female ratio of patients enrolled	1.2	1.1	1.4	1.2
Malaria microscopy positive, n (% patients enrolled)	452 (69.8)	176 (47.9)	149 (46.0)	777 (58.0)
Severe malaria, n (% of patients enrolled)	54 (8.3)	81 (22.1)	117 (36.1)	252 (18.8)
BSI (episodes), n (% of enrolled)	31 (4.8)	39 (10.6)	48 (14.8)	118 (8.8)
Severe malaria combined with BSI, n (% of patients enrolled)	1 (0.2)	4 (1.1)	1 (0.3)	5 (0.4)
All malaria combined with BSI, n (% of patients enrolled)	14 (2.2)	7 (1.9)	1 (0.3)	22 (1.6)
Acute severe malnutrition, n (% of patients enrolled)	12 (1.8)	74 (20.2)	70 (21.6)	156 (11.6)
Previous antimalarial use, n (% of patients enrolled)	50 (7.7)	138 (37.6)	162 (50.0)	350 (26.1)
Previous antibiotic use, n (% of patients enrolled)	27 (4.2)	103 (28.1)	122 (37.6)	252 (18.8)

HDSS: Health and demographic surveillance system. HC: Healthcare Centre. CMA: Centre Médical avec Antenne Chirurgical

### Bloodstream infections, invasive salmonellosis and severe malaria

Blood culture confirmed BSI was found in 8.8% of patients (Table 1); there were no recurrent episodes of BSI. Among the 118 BSI episodes, 120 pathogens were isolated (Table 2). Invasive salmonellosis represented over two-thirds (83/118, 70.3%) of BSI episodes, with *Salmonella* Typhi and iNTS accounting for 13.6% and 56.8% of BSI episodes and representing 16 and 67 isolates respectively.

**Table 2:** Breakdown of all pathogens isolated for the total study area and the two study sites in the HDSS catchment area.

	Patients from HDSS catchment area		Patients from outside HDSS area	Total
	HC Nazoanga	CMA	CMA	
BSI (episodes), n	31	39	48	118^a^
*Salmonella* Typhi, n (% of BSI episodes)	7 (22.6)	5 (12.8)	4 (8.3)	16 (13.6)
*Salmonella* non-Typhi, n (% of BSI episodes)	16 (51.6)	23 (59.0)	28 (58.3)	67 (56.8)
*Salmonella* Typhimurium n (% of *Salmonella* non-Typhi)	15 (93.8)	16 (69.6)	17 (60.7)	48 (71.6)
*Salmonella* Enteritidis, n (% of *Salmonella* non-Typhi)	1 (6.2)	5 (21.7)	11 (39.3)	17 (25.4)
*Salmonella* Brancaster, n (% of *Salmonella* non-Typhi)	0	1 (4.3)	0	1 (1.5)
*Salmonella* Freetown, n (% of *Salmonella* non-Typhi)	0	1 (4.3)	0	1 (1.5)
*Streptococcus pneumoniae*, n (% of BSI episodes)	3 (9.7)	5 (12.8)	7 (14.6)	15 (12.7)
*Staphylococcus aureus*, n (% of BSI episodes)	2 (6.4)	1 (2.6)	4 (8.3)	7 (5.9)
*Escherichia coli*, n (% of BSI episodes)	1 (3.2)	3 (7.7)	3 (6.2)	7 (5.9)
*Haemophilus influenzae*, n (% of BSI episodes)	1 (3.2)	1 (2.6)	1 (2.1)	3 (2.5)
*Neisseria meningitidis W135*, n (% of BSI episodes)	1 (3.2)	0	0	1 (0.8)
*Streptococcus* group A, n (% of BSI episodes)	0	1 (2.6)	1 (2.1)	2 (1.7)
*Pseudomonas aeruginosa*, n (% of BSI episodes)	0	1 (2.6)	0	1 (0.8)
*Enterobacter spp*, n (% of BSI episodes)	0	0	1 (2.1)	1 (0.8)

HDSS: Health and demographic surveillance system. HC: Healthcare Centre. CMA: Centre Médical avec Antenne Chirurgicale

^a^ Two bloodstream infection (BSI) episodes yielded each two pathogens, resulting in 120 pathogens.

Two *Salmonella* non-Typhi isolates were serotyped as *Salmonella* Brancaster and *Salmonella* Freetown.

Overall, 58.0% (777/1,339) of children had microscopically confirmed malaria, of whom 32.4% (252/777, 18.8% of children enrolled) had severe malaria. All malaria cases were microscopically confirmed as *Plasmodium falciparum* malaria. Among the patients for whom both RDT and microscopy were performed, 11.7% (76/648) had a positive RDT but a negative result for microscopy, which is indicative of a recent malaria infection.

A total of 2.8% (22/777) of patients with malaria also had a BSI. Conversely, 18.6% (22/118) of patients with BSI had malaria. The pathogens found in those patients were iNTS (n = 12) *Salmonella* Typhi (n = 3), *Streptococcus pneumoniae* (n = 5) and *Staphylococcus aureus* (n = 2).

Children with reported use of antibiotics prior to sampling had a higher proportion of growth compared to those without antibiotics (13.9% (35/252) versus 7.8% (85/1,087) respectively p = 0.001). There was no apparent difference between pathogens recovered in both groups.

In-hospital mortality rates were 10.2% (12/118), 6.0% (4/67) and 7.1% (18/252) for BSI, iNTS and severe malaria respectively; none of the children with *Salmonella* Typhi BSI died.

### Crude and adjusted incidence: Correction factors

**Fig 1:**
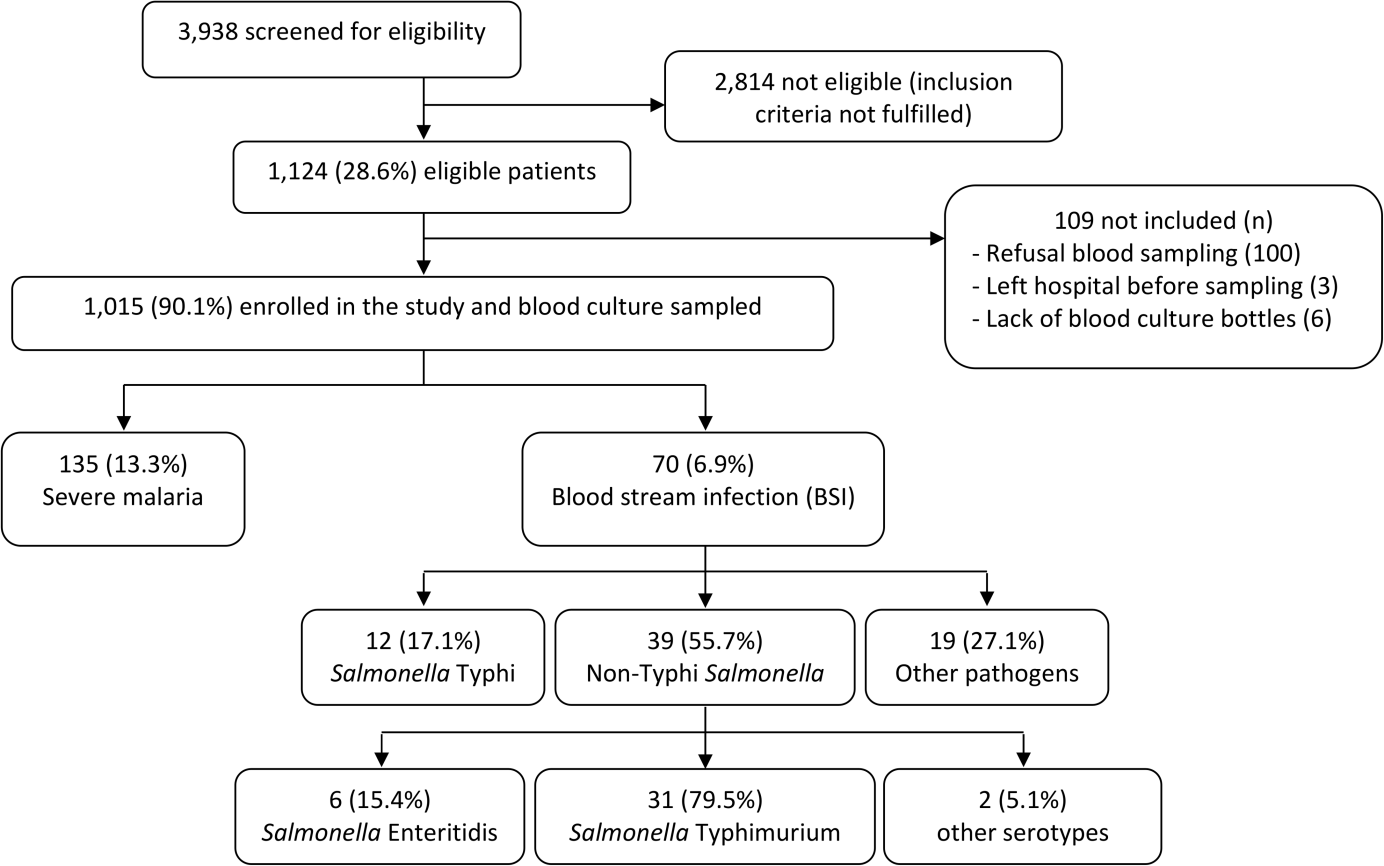
Patients’ flow for the patients within the HDSS catchment area and for both inclusion sites. One grown blood culture contained 2 isolates.

Crude and adjusted incidence rates were calculated based on a one year time frame: from May 2013 to May 2014. Only patients living inside the HDSS area were considered. Fig 1 lists the study flow for this subgroup of patients. The applied correction factors were consecutively (i) x 4 for non-referral, (ii) x 2 for seeking healthcare elsewhere than in CMA or Health Centers, (iii) x 1.1 for non-enrollment of eligible children, (iv) x 2.4 and x 2.2 for the < 5 years old group and the 5–15 years old group respectively to correct for both the known sensitivity of blood cultures and under-filling of blood culture bottles.

(i) Non-referral: Data from the District Health Officer showed that a median of 25% of patients with fever are referred from HCs to CMA. The surveillance data showed that a large proportion (>80%) of patients with confirmed BSI were not referred from HC Nazoanga to CMA, and supported the correction factor based on District Health Office data of 1/0.25 = 4.0. This correction factor was only applied to patients who were referred from other HCs than Nazoanga. For example, in the age group < 5 years, there were a total of 38 children with iNTS BSI; 12 of them were referred to CMA from Healthcare Centers other than HC Nazoanga, the remaining 26 were admitted to CMA without referral or were enrolled at HC Nazoanga. In this case, application of the correction factor resulted in a total of 74 BSI episodes: The correction factor of 4 was applied to the 12 cases of iNTS referred from other HC than Nazoanga (4 x 12 = 48), plus 26 enrolled at HC Nazoanga or directly at CMA. Among the age group 5–15 years, there were no patients with an iNTS BSI so for this age group this correction factor was not applied.

(ii) Seeking healthcare elsewhere than in CMA or Health Centers: A total of 164 households were visited during the HUS, representing 1,615 participants including 695 children < 15 years old. Among children a total of 789 episodes of febrile illness were reported over a total observation period of 6,957 months. After extrapolating this data to represent a 12-months period for each participant, a total of 920 episodes of febrile illness in children were recorded over 8,340 months; In 460 (50%) of episodes healthcare was sought elsewhere than in Health Centers or CMA. A corresponding correction factor of 1/0.5 = x 2.0 was applied.

(iii) Non-enrollment of eligible children. The proportion of children eligible but not enrolled was 9.7% (Fig 1), the corresponding correction factor was 1/0.097 = x 1.1.

(iv) Sensitivity of the blood cultures and inadequate filling. The volume lost by under-filling was 0.4 ml for both age groups (the minimum volume required (1 ml) minus the actual median volume of under-filled bottles (= 0.6 ml)). This volume was subsequently multiplied by the proportion of under-filled bottles in each age group. For age group < 5 years this was 39.8% and for age group 5–15 years this was 24.9%, resulting in a loss of 0.16 ml and 0.10 ml respectively. Corresponding correction factors were 1/ (1–0.16) = x 1.2 and 1/ (1–0.10) = x 1.1 respectively. Combined with the correction factor for the 50% estimated overall sensitivity of blood cultures, the final correction factors were 1.2 x 2 = x 2.4 and 1.1 x 2 = x 2.2 for age groups < 5 years and 5–15 years respectively.

### Crude and adjusted incidence rates for bloodstream infections and severe malaria

Table 3 shows the crude and adjusted incidence rates for BSI and severe malaria in the HDSS area. In the < 5 years old group, adjusted incidence rates of BSI caused by all pathogens and by iNTS were 6,374 and 4,138 per 100,000 PYO respectively. The incidence rate of severe malaria in the < 5 years old group was 2,866 per 100,000 PYO. In the 5–15 years old group, incidence rates for all BSI, iNTS and severe malaria were more than a 10-fold lower than in the < 5 years old group (397, 25 and 135 per 100,000 PYO respectively) with the highest difference observed for iNTS; in contrast, the incidence for *Salmonella* Typhi BSI was similar to that observed in the < 5 years old group (224 and 273 per 100,000 PYO respectively). Of note, nearly three-quarter (73.8% (45/61)) of iNTS isolates of the < 5 years old group were recovered in children between 2 months and 3 years of age. There was no association with severe acute malnutrition. Reported data are made available in S1 Database.

**Table 3:** Crude and adjusted incidence rates of bloodstream infection and severe malaria for children from HDSS area.

Age groups (years)	Nrs	Crude Incidence	Nrs of BSI (not corrected for referral)^a^	Incidence adjusted for non-referral	Incidence adjusted for health seeking behavior (x 2.0)	Incidence adjusted for non-enrollment (x 1.1)	Incidence adjusted for blood culture sensitivity and filling rate (x 2.4 (< 5 years old group) and x 2.2 (5–15 years old group)
**All bloodstream infection pathogens**							
< 5	54	572	114	1,207	2,414	2,656	**6,374**
		(430–746)					(5,890–6,892)
5–15	16	82	16	82	164	180	**397**
		(47–133)					(311–493)
***Salmonella* Typhi bloodstream infection**							
< 5	1	11	4	42	85	93	**224**
		(1–59)					(138–340)
5–15	11	56	11	56	113	124	**273**
		(28–101)					(203–355)
**non-Typhoidal *Salmonella* bloodstream infection**							
< 5	38	402	74	784	1,567	1,724	**4,138**
		(285–552)					(3,740–4,572)
5–15	1	5	1	5	10	11	**25**
		(1–29)					(8–60)
**Severe malaria**							
< 5	123	1303	NA	NA	2,605	**2,866**	NA
		(1,083–1,554)				(2,538–3,233)	
5–15	12	61	NA	NA	123	**135**	NA
		(32–107)				(87–195)	

Incidences are expressed as numbers/100,000 person observations years (PYO); 95% confidence intervals are written within brackets. BSI: bloodstream infections. NA: non-applicable. Nrs: numbers. iNTS: invasive non-Typhoidal *Salmonella*.

^a^ Correction was applied for those BSI that were referred from HC other than Nazoanga.

### Seasonality and geography

**Fig 2:**
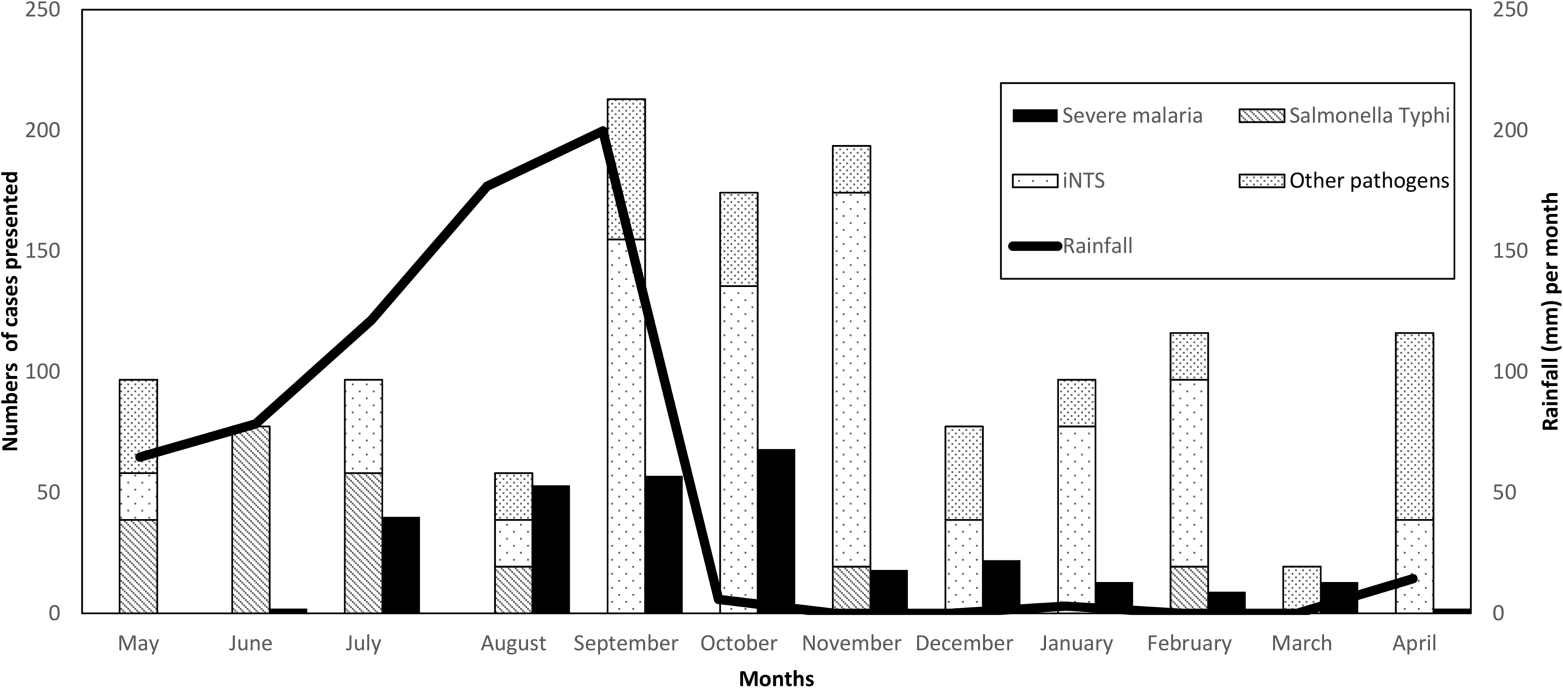
Distribution of monthly rainfall in relation to numbers of severe malaria and bloodstream infections for the patients from the HDSS zone. For comparison, the adjusted numbers of cases were used. HDSS: Health and Demographic Surveillance System. iNTS: invasive Non Typhoid Salmonellosis.

Fig 2 shows the monthly distribution of rainfall, severe malaria and BSI, including *Salmonella* Typhi and iNTS. The number of iNTS sharply increased sharply in September and lasted for a period of six months. The increase coincided with the peak of the rainy season and the seasonal increase of *Plasmodium falciparum* malaria. There were no apparent clusters (in time and location) for iNTS [11]. In our cohort we found that 6/16 *Salmonella* Typhi isolates were recovered from patients from Nazoanga village over a three-month period, which may be suggestive of a common source outbreak.

## Discussion

The present study in rural Burkina Faso assessed the population-based incidence of *Salmonella* BSI among febrile and severely ill children in a rural area with seasonal malaria transmission.

*Salmonella* Typhi and iNTS accounted for 13.3% and 55.8% of all 118 BSI episodes; 71.6% of iNTS (48/67) isolates were *Salmonella* Typhimurium and 25.4% (17/67) *Salmonella* Enteritidis; there was no apparent geographical clustering. The adjusted incidence of iNTS in the < 5 years old group was 4,138 per 100,000 PYO and exceeded the incidence of severe malaria (2,866 per 100,000 PYO). Incidences of *Salmonella* Typhi BSI were 224 and 273 per 1000,000 PYO for the < 5 years old group and 5–15 years old group respectively. Most iNTS occurred at the peak of the rainy season and during a three-month period thereafter. There was no clear-cut seasonal pattern for *Salmonella* Typhi.

### Limitations

Limitations to this study included the relatively short duration of the study. The study period of 12 months was chosen to adjust for seasonal variation. Additionally, the absolute numbers of iNTS and *Salmonella* Typhi cases among the 5–15 years and <5 years old groups respectively were relatively low, resulting in corresponding incidence rates with wide confidence intervals. The proportion of iNTS and Salmonella Typhi grown from blood cultures in the present study were however in line with those of a similar study conducted at the same site one year previously [11].

Despite training and supervision subjectivity in clinical assessment (and consequent differences in blood culture sampling) cannot be excluded. Other limitations of the present study are related to refusal rates, sub-optimal volumes taken for blood culture and the organization of the HUS. The overall refusal rate among eligible children was 9.4%, of which the majority occurred over a short period at the beginning of the study. The adjusted incidence rates were corrected for refusals. The common practice of sampling sub-optimal volumes for blood culture was related to sampling in small children [17] and for which a correction factor was applied. Due to constraints of logistics and accessibility (rainy season), the HUS could not be organized at all selected household sites at all intended intervals, which may have affected the HUS results.

### Comparison of the incidence of invasive salmonella BSI with other studies from sub-Saharan Africa

Only few studies have provided population-based incidence rates for BSI caused by *Salmonella* Typhi [18–21] or iNTS [20–23] and their methods differed from our study, particularly with regard to patient selection and correction factors used for converting crude to adjusted incidence rates (Tables 4 and 5).

**Table 4:** Studies from rural and urban sub-Saharan Africa with adjusted incidence rates of invasive *Salmonella* Typhi infection.

Country, urban versus rural setting, year of study	Design	Age group	Adjusted incidence per 100,000 PYO (95% CI)	Factors used to adjust
***Salmonella* Typhi**				
Ghana, rural (Agogo), 2009 [20]	Prospective hospital based	< 5 yrs	330 (180–490)	• Health seeking behavior • Non-enrollment
Kenya, rural (Lwak), 2009 [18]	Population-based surveillance	2–4 yrs	743 (116–1,804)	• Health seeking behavior • Non-enrollment
		5–9 yrs	216 (56–903)	
Kenya, urban (Kibera), 2009 [18]	Population-based surveillance	2–4 yrs	2,243 (1,589–3,171)	
		5–9 yrs	1,788 (1,348–2,373)	
Tanzania, rural (Pemba, Zanzibar), 2010 [19]	Population-based surveillance	< 5 yrs	84 (69–101)	• Health seeking behavior • Non-enrollment • Blood culture filling rate • Blood culture sensitivity
		5–15 yrs	101 (86–121)	
Burkina Faso, semi-urban, (Polesgo), 2017 [21]	Population-based surveillance	0–1 yrs	0 (0–0)	• Health seeking behavior • Non-enrollment
		2–4 yrs	1890 (1202–2972)	
		5–14 yrs	485 (263–896)	
		< 15 yrs	719 (500–1035)	
Burkina Faso, semi-urban, (Nionko II), 2017 [21]	Population-based surveillance	0–1 yrs	0 (0–0)	
		2–4 yrs	251 (107–590)	
		5–14 yrs	315 (191–519)	
		< 15 yrs	227 (148–350)	

PYO: person years of observation. yrs: years of age.

**Table 5:** Studies from rural and urban sub-Saharan Africa with adjusted incidence rates of invasive Non-Typhoidal *Salmonella* infection.

Country, urban versus rural setting, year of study	Design	Age group	Adjusted incidence per 100,000 PYO (95% CI)	Factors used to adjust	Malaria, HIV and malnutrition prevalence
**Non-Typhoidal *Salmonella***					
Ghana, rural (Agogo), 2009 [20]	Prospective hospital based	< 5 yrs	2,520 (2,110–2,940)	• Health seeking behavior • Non-enrollment	• Malaria holoendemic • HIV 1.8% (adults) • Malnutrition (23%)
Kenya, rural (Asembo), 2009 [22]	Population-based surveillance	< 5 yrs	2,085 (1,181–2,990)	• Health seeking behavior • Non-enrollment	• Malaria endemic • HIV adults (15–17%)
Kenya, urban (Kibera), 2009 [22]	Population-based surveillance	< 5 yrs	260 (102–419)		
Kenya, Rural (Kilifi), 2014 [23]	Retrospective population based surveillance	< 5 yrs	33 (28–38)	• Non-enrollment • Blood culture contamination	• Malaria holoendemic • HIV adults (3.3%)
		5–15 yrs	2 (1–4)		
Burkina Faso, semi-urban, (Polesgo), 2017 [21]	Population-based surveillance	0–1 yrs	431 (162–1147)	• Health seeking behavior • Non-enrollment	• Malaria holoendemic • HIV adults (0.9%)
		2–4 yrs	630 (288–1380)		
		5–14 yrs	0 (0–0)		
		< 15 yrs	255 (138–470)		
Burkina Faso, semi-urban (Nionko II), 2017 [21]	Population-based surveillance	0–1 yrs	753 (460–1233)		
		2–4 yrs	753 (460–1233)		
		5–14 yrs	236 (133–640)		
		< 15 yrs	475 (352–640)		

PYO: person years of observation. yrs: years of age.

Incidence rates of *Salmonella* Typhi were two-to-three fold higher in the present study compared to those observed in Pemba, an island of the Zanzibar archipelago [19]. Differences may partly be due to not correcting for referral rate in the latter study. Compared to studies from rural Ghana and Kenya the present incidence rates were lower [18, 20]. In urban Kenya (Kibera) the incidence rates were 10-fold higher compared to the present ones, which may be attributed to the high population density at the Kenyan site (71,000 people/km^2^) [18]. Marks et al [21] recently published a multicentre study performed on 13 sites in different sub-Saharan African countries, including two semi-urban sites in Burkina Faso. Their study demonstrated a higher rate of *Salmonella* Typhi infection in both sites compared to the present one, which may be related to the higher population density at the semi-urban sites (2204/km^2^ and 5163/km^2^ in Nioko and Polesgo respectively, compared to 100/km^2^ in Nanoro).

In the present study incidence rates of iNTS among the < 5 years old group were nearly twice as high compared to observed incidences rates from rural Kenya and Ghana [20, 22]. This difference can be partly explained by differences in applied correction factors: both studies did not correct for blood culture sensitivity and filling rate. Although speculative, adjusting for these factors as done in the present study would have resulted in higher incidences than found in the present study at both the Kenyan and the Ghanian site. At the Ghanian site, this speculative higher incidence might be partly explained by a higher proportion of malnutrition (23%) compared to the present study (11%) and by the fact that the study was exclusively hospital-based, skewing the results to the most severe cases [20].

By contrast, another study site in rural Kenya (Kilifi) reported an incidence of only 33/100,000 PYO among the < 5 years old group; the authors mentioned a declining incidence of *P*. *falciparum* malaria during the study period as a factor explaining this low incidence [23, 24]. The only study carried out in an urban (slum) setting (Kibera, Kenya) notably reported a 10 to 20-fold lower incidence rate (260/100,000 PYO) compared to the rural sites in Ghana, Kenya as well as the present study site [22]. The Typhoid Fever Surveillance in Africa Program consortium (TSAP) [21] reported significantly lower incidence rates for iNTS in both Burkina sites than the current study. This may be explained by the difference in applied correction factors. In particular, the TSAP study did not correct for blood culture sensitivity and filling rates. Another explanation may be the difference between study site setting (semi-urban in the TSAP study versus rural in the present one). In can be remarked that the highest incidence of iNTS in the TSAP study was observed in a rural setting in Ghana. One possible explanation for the difference in prevalence of iNTS in difference may be malnutrition [25, 26].

### Application of correction factors, comparison to literature

Most correction factors used for incidence calculation in this study have been previously described. Unlike previous studies, the current study additionally corrected for non-referral of patients from healthcare centers to the reference hospital. Non-referral was estimated by statistics obtained by the district health office, but could not be validated. As a result, this correction factor may have caused either an over- or underestimation of the true incidence. The correction factor was applied to a limited number of cases and we therefore believe has had a low impact on the incidence calculations.

The HUS formed an integral part of the current study and was organized in the same way as previously reported by the TSAP consortium. The HUS was corrected for seasonality by covering a full year for each sampled household. We would however like to caution that health-seeking behavior may slightly differ over age group and gender. The present study did not correct for these factor because of concern to decrease reliability as numbers of cases in some groups would be small. Surprisingly, many other authors do not correct for blood culture nor filling rate, a factor of high impact to sensitivity. This finding is further supported by Gorden et al. [27] who demonstrated that the yield of *Salmonella* Typhi from blood culture was 40.3% compared to 86.7% from bone marrow punction among HIV infected adults in Malawi.

### Distribution of non-Typhoidal Salmonella serotypes in sub-Saharan Africa

*Salmonella* Typhimurium and *Salmonella* Enteritidis were the most prevalent serotypes at proportions of 71.6% and 25.4% respectively. These proportions are in line with the results of a meta-analysis of community acquired BSI in children in Africa which showed that serotypes Typhimurium and Enteritidis accounted for respectively two-thirds (65.2%) and one third (33.1%) of 706 *Salmonella* isolates [28].

### Comparison of seasonality and geography, relation with P. falciparum malaria

We saw an increase of iNTS at the peak of the rainy season, extending into the dry season (Fig 2). Annual peaks of iNTS and malaria coincide [29, 30] and rainfall may relate to both the intensity of malaria transmission and risk of iNTS infection [31, 32]. Severe *P*. *falciparum* malaria is moreover thought to predispose to iNTS in several ways: (i) iron released by lysis of *P*. *falciparum*-infected red blood cells is an essential substrate for bacteria; (ii) the accumulation of the hemozoin inside monocytes alters cell differentiation to antigen presenting cells; (iii) production of interleukin-12 during malaria infection may increase susceptibility to iNTS; (iv) sequestration of *P*. *falciparum* parasites in the microcirculation of the intestinal mucosa during sever malaria renders it more susceptible to bacteria that colonize the gut [33–36]. The apparent increase of *Salmonella* Typhi at the start of the rainy season was linked to Nazoanga village and, may have represented clustered cases from a common source outbreak.

### Co-infections between P. falciparum malaria and bloodstream infections

In the present study, combined malaria (uncomplicated and severe) and BSI was observed in 2.8% of children with malaria. The overall percentage of BSI among children with malaria was lower than the 5.6% to 6.5% proportion aggregated in two metadata studies on BSI in Africa [28, 37]. There is no obvious explanation for the lower proportion of BSI among children with malaria in the present study, although it is consistent with that of a previous study conducted in the same area [11].

### Relevance of present findings

The present findings depict *Salmonella* Typhi and iNTS as major public health issues in children from West Africa. As for *Salmonella* Typhi, the present incidence confirms that West Africa may be considered among the high-incidence regions (> 100 cases/100,000/year) [38]. This contrasts to estimations from a decade ago, when typhoid fever in Africa was categorized as medium incidence (*i*.*e*. 10–100 cases/100,000/year), as opposed to high incidence regions in South-Central and South-East Asia [39]. This switch probably does not reflect an actual increase in cases but rather may be ascribed to a previous under-estimation due to the lack of microbiologically documented studies [38].

Given their association with multidrug resistance and case fatality [3, 40, 41], and the fact that their reservoir and transmission are still poorly understood, iNTS are urgent candidates for vaccine development [42]. Proof-of-principle studies from animal models are available for vaccine prototypes targeting O-antigens, flagellin proteins, and other outer membrane proteins of the Typhimurium and Enteritidis serotypes. Moreover, new glycoconjugate vaccines against *Salmonella* (both *Salmonella* Typhi and non-typhoidal *Salmonella* serotypes) in development include live-attenuated, protein-based vaccines with a novel self-adjuvanting antigen-delivery strategy (the so-called Generalized Modules for Membrane Antigens (GMMA) technology) vaccines [43]. An additional advantage is that iNTS vaccines fit the age groups targeted by the current Expanded Program of Immunization in sub-Saharan Africa, thereby assuring logistical feasibility of mass vaccination.

## Conclusions

The present results confirm iNTS as the predominant BSI pathogen in children in rural Africa [44]. It shows that the combined case load of invasive *Salmonella* Typhi and *Salmonella* non-Typhi infection was higher than that of severe malaria in children across different ages in rural Burkina Faso. Even though malaria has been declining worldwide, it is clear that the incidence of bloodstream infection and invasive salmonellosis in sub-Saharan Africa has been underestimated before. This is probably due to the lack of appropriate diagnostic methods to obtain accurate bacteriological diagnosis, in particular in low resource settings where malaria is most prevalent. Significant efforts are still necessary towards better preventive, diagnostic and therapeutic tools to improve the clinical care and community control of this neglected disease. Prioritization of the implementation of *Salmonella* Typhi as well as iNTS vaccines is needed.

## Supporting information

S1 Appendix Quality indicators. (DOCX)

S2 Appendix Methodology of applied correction factors. (DOCX)

S1 Database Study database stripped of patient indicators. Tab one: database/ Tab 2: code.
(XLSX)
