# Supplementary material for: Population-based incidence, seasonality and serotype distribution of invasive salmonellosis among children in Nanoro, rural Burkina Faso
Source: PLoS One. 2017 Jul 10;12(7):e0178577. doi: 10.1371/journal.pone.0178577 (PMC5503169; doi:10.1371/journal.pone.0178577)
Supplement: S1 Appendix — (DOCX) [file pone.0178577.s001.docx]

**S1 Appendix. Quality indicators**

The overall % of contaminants in blood cultures was 1.5 %; proportions of contaminants were similar at both sampling sites and constant over time. A total of 1,238 (79.3 %) blood culture bottles had been weighed before and after sampling. Overall, 51.6 % of bottles was correctly filled, 11.1 % and 37.3 % of bottles were over- and under-filled respectively. Median (Interquartile range, IQR) blood volume was 1.2 (0.7 – 2.1) ml. Proportions of under-filled blood culture bottles were 39.8 % and 24.9 % among the < 5 years and 5 – 15 years age groups respectively, with corresponding median volumes (IQR) of 0.6 ml (0.3 – 0.8 ml) and 0.6 ml (0.4 – 0.8 ml) respectively. Under-filling of bottles occurred more in HC Nazoanga than in CMA (350 / 630 (58 %) versus 112 / 635 (17.6 %), p < 0.001). Growth of pathogens tended to be higher among correctly filled and over-filled blood cultures (74/776, 9.5 %) versus under-filled blood cultures (31/462, 6.7 %) (p = 0.087). The proportions of contaminants in the under-filled bottles versus the correctly filled and over-filled bottles were (1.7 %, 8/462) versus (0.9 %, 7/776) (p = 0.21). The maximum transport time between HC Nazoanga and CRUN was 1h 36 min.
