# Supplementary material for: Population-based incidence, seasonality and serotype distribution of invasive salmonellosis among children in Nanoro, rural Burkina Faso
Source: PLoS One. 2017 Jul 10;12(7):e0178577. doi: 10.1371/journal.pone.0178577 (PMC5503169; doi:10.1371/journal.pone.0178577)
Supplement: S2 Appendix — (DOCX) [file pone.0178577.s002.docx]

**S2 Appendix. Methodology of applied correction factors.**

**Methodology of the HUS**

A Healthcare Utilization Survey (HUS) was carried out in the HDSS area in order to map the healthcare utilization of children in case of fever. The sample size was calculated as 400 subjects, presuming that 50 % of subjects would indicate that they would seek care, and with a 5 % precision. Assuming a design effect of 2 (due to clustering of data within households) and presuming five subjects in each household, a sample size of 160 households representing 800 subjects was required. Each household was visited one to four times over a period of 18 months. The head of household was asked about the number of episodes of fever that had occurred in each household member during the previous three months and about the corresponding health seeking behavior. Health seeking behavior was categorized as follows: 1) stayed at home, 2) went to a pharmacist, 3) went to a traditional healer, 4) went to a Health Center, 5) went to or was referred to CMA, 6) went to other types of healthcare. Categories 1, 2, 3 and 6 were grouped together as “seeking healthcare elsewhere than CMA and Health Centers” and were not available for enrollment. Surveys were performed as part of the HDSS routine visits. In some parts of the HDSS catchment area two surveys were performed within a 2-month interval; in these cases the results from one of the surveys were not considered to prevent overlap in data. Results of the three remaining surveys were extrapolated to represent a 12-month period. Data obtained from households that were visited less than four times were also extrapolated to represent a 12-month period. For analysis only the data from children < 15 years old was considered.

A total of 164 households were visited, representing 1,615 subjects including 695 children < 15 years old. In children a total of 789 episodes of febrile illness were reported over a total observation period of 6,957 months. After extrapolating this data to represent a 12-months period for each participant, a total of 920 episodes of febrile illness in children were recorded over 8,340 months; healthcare elsewhere than in Health Centers or CMA or was sought in 460 (50 %) episodes. A corresponding correction factor of 1/0.5 = x 2.0 was applied.

**Sensitivity of the blood cultures and inadequate filling**

The yield of blood cultures is mainly related to the volume of blood sampled, (apart from other factors such as intermittent presence of bacteria in the blood and the use of antibiotics prior to sampling). In adults and in the case of severe sepsis, overall sensitivity of blood cultures has been estimated at < 50 % [36]. Further evidence to support this estimation has been obtained for instance by Bayesian latent class methods in the case of melioidosis (sensitivity estimated at 62 % [37]) and by comparison with bone marrow culture in the case of *Salmonella* Typhi (40.3 – 86.7 % [31]). Extrapolation of these data to children is presumptive [12]. In the case of BSI, children generally have higher bacterial loads compared to adults, (except in the case of so-called occult bacteremia) [12] but there are no data about the numbers of bacteria in the blood during iNTS BSI. We set the overall blood culture sensitivity at 50 %, in line with estimations for adults.

Under-filling and over-filling of blood cultures was defined as below or above the recommended volume of 1 - 3 ml [38]. To estimate the impact of under-filling, the volume lost by under-filling was calculated by subtracting the actual median volume of the under-filled bottles from the minimum volume required (= 1.0 ml). Next, this difference was multiplied by the proportion of under-filled bottles and quantified. The age groups of < 5 years old and 5 – 15 years old were considered separately [12]. The volume lost by under-filling was 0.4 ml for both age groups (= minimum volume required of 1 ml minus the actual median volume of under-filled bottles (= 0.6 ml)). Supplement 2 provides details of the median volume in each age group. This volume was subsequently multiplied by the proportion of under-filled bottles in the age groups < 5 years (39.8 %) and 5 – 15 years (24. 9%), resulting in a loss of 0.16 ml and 0.10 ml respectively. Corresponding correction factors 1/ (1 – 0.16) = x 1.2 and 1/ (1 – 0.10) = x 1.1 respectively. Combined with the correction factor for the 50 % estimated overall sensitivity of blood cultures, the final correction factors were 1.2 x 2 = x 2.4 and 1.1 x 2 = x 2.2 for age groups < 5 years and 5 – 15 years respectively.
